# Parsimony and the rank of a flattening matrix

**DOI:** 10.1007/s00285-023-01875-y

**Published:** 2023-02-09

**Authors:** Jandre Snyman, Colin Fox, David Bryant

**Affiliations:** 1 Department of Mathematics and Statistics, University of Otago, Dunedin, New Zealand; 2 Department of Physics, University of Otago, Dunedin, New Zealand

**Keywords:** Phylogeny, Tensor flattening, Rank formula, Markov models on trees, Phylogenetic invariants, 92D15, 60J10, 15A69

## Abstract

The standard models of sequence evolution on a tree determine probabilities for every character or site pattern. A flattening is an arrangement of these probabilities into a matrix, with rows corresponding to all possible site patterns for one set *A* of taxa and columns corresponding to all site patterns for another set *B* of taxa. Flattenings have been used to prove difficult results relating to phylogenetic invariants and consistency and also form the basis of several methods of phylogenetic inference. We prove that the rank of the flattening equals $r^{\nu _T(A|B)}$, where *r* is the number of states and $\nu _T(A|B)$ is the minimum size of a vertex cut separating *A* from *B*. When *T* is binary the rank of the flattening equals $r^{\ell _T(A|B)}$ where $\ell _T(A|B)$ equals the parsimony length of the binary character separating *A* and *B*. We provide a direct proof that requires little more than undergraduate algebra, but note that the formula could also be derived from work by Casanellas and Fernández-Sánchez (2011) on phylogenetic invariants.

## Introduction

Behind any statistical inference in phylogenetics is a model describing the evolution of the states (alleles/nucleotides/amino acids) observed at each site in the alignment. Under the standard models, the distribution of states at a site is determined by three types of parameters: the phylogeny itself, the distribution of the state at the root, and the transition probabilities along each edge. Together these generate the joint distribution for the state at each leaf, which in turn corresponds to a column of the alignment (reviewed in Bryant et al. (2005), Felsenstein (2004)).

**Fig. 1:**
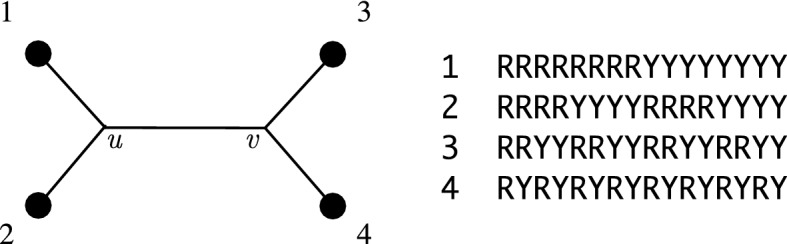
An example of patterns or sites on a four taxa tree. The 16 possible patterns are listed in the table on the right - any one of these could be the pattern for a particular site

An assignment of states to the taxa is often called a *pattern*. If there are *r* states (say $r=2$ for binary, $r=4$ for nucleotides, $r=20$ for amino acids) and *n* taxa then there are $r^n$ possible patterns. In the example in Fig.  1 we have $r=2$ and $n=4$, so $r^n = 16$ possible pattern for the joint distribution. The two states here code for *purines* (coded $\texttt{R}$, standing for nucleotides *A* or *G*) and *pyrimidines* (coded $\texttt{Y}$, standing for *C* or *T*). Let $p_{i_1i_2i_3i_4}$ denote the joint probability of pattern $i_1i_2i_3i_4$, that is the probability under the model of observing state $i_1$ at leaf 1, $i_2$ at leaf 2 and so on. We can think of these as elements of an $r^n$ dimensional vector

1 $$\begin{aligned} \left[ \begin{matrix}p_{\tiny {\texttt{R}}\tiny {\texttt{R}}\tiny {\texttt{R}}\tiny {\texttt{R}}}\\ p_{\tiny {\texttt{R}}\tiny {\texttt{R}}\tiny {\texttt{R}}\tiny {\texttt{Y}}}\\ p_{\tiny {\texttt{R}}\tiny {\texttt{R}}\tiny {\texttt{Y}}\tiny {\texttt{R}}}\\ \vdots \\ p_{\tiny {\texttt{Y}}\tiny {\texttt{Y}}\tiny {\texttt{Y}}\tiny {\texttt{Y}}}\end{matrix}\right] \end{aligned}$$

or as a $2 \times 2 \times 2 \times 2$ multidimensional array or tensor. Alternatively we can reshape the vector into a matrix, such as

2 $$\begin{aligned} \left[ \begin{matrix}p_{\tiny {\texttt{R}}\tiny {\texttt{R}}\tiny {\texttt{R}}\tiny {\texttt{R}}} &{} p_{\tiny {\texttt{R}}\tiny {\texttt{R}}\tiny {\texttt{R}}\tiny {\texttt{Y}}} &{} p_{\tiny {\texttt{R}}\tiny {\texttt{R}}\tiny {\texttt{Y}}\tiny {\texttt{R}}} &{} p_{\tiny {\texttt{R}}\tiny {\texttt{R}}\tiny {\texttt{Y}}\tiny {\texttt{Y}}} \\ p_{\tiny {\texttt{R}}\tiny {\texttt{Y}}\tiny {\texttt{R}}\tiny {\texttt{R}}} &{} p_{\tiny {\texttt{R}}\tiny {\texttt{Y}}\tiny {\texttt{R}}\tiny {\texttt{Y}}} &{} p_{\tiny {\texttt{R}}\tiny {\texttt{Y}}\tiny {\texttt{Y}}\tiny {\texttt{R}}} &{} p_{\tiny {\texttt{R}}\tiny {\texttt{Y}}\tiny {\texttt{Y}}\tiny {\texttt{Y}}} \\ p_{\tiny {\texttt{Y}}\tiny {\texttt{R}}\tiny {\texttt{R}}\tiny {\texttt{R}}} &{} p_{\tiny {\texttt{Y}}\tiny {\texttt{R}}\tiny {\texttt{R}}\tiny {\texttt{Y}}} &{} p_{\tiny {\texttt{Y}}\tiny {\texttt{R}}\tiny {\texttt{Y}}\tiny {\texttt{R}}} &{} p_{\tiny {\texttt{Y}}\tiny {\texttt{R}}\tiny {\texttt{Y}}\tiny {\texttt{Y}}} \\ p_{\tiny {\texttt{Y}}\tiny {\texttt{Y}}\tiny {\texttt{R}}\tiny {\texttt{R}}} &{} p_{\tiny {\texttt{Y}}\tiny {\texttt{Y}}\tiny {\texttt{R}}\tiny {\texttt{Y}}} &{} p_{\tiny {\texttt{Y}}\tiny {\texttt{Y}}\tiny {\texttt{Y}}\tiny {\texttt{R}}} &{} p_{\tiny {\texttt{Y}}\tiny {\texttt{Y}}\tiny {\texttt{Y}}\tiny {\texttt{Y}}} \end{matrix}\right] . \end{aligned}$$

In this matrix, the rows correspond to the $r^2$ possible ways of assigning states to taxa 1 and 2 while the columns correspond to the $r^2$ possible ways of assigning states to taxa 3 and 4. A matrix of this form is called a *flattening*. In tensor terminology, the flattening is an example of an *unfolding* of the tensor of pattern probabilities. The idea was introduced into phylogenetics by Pachter and Sturmfels (2004) and developed extensively by Allman and Rhodes to solve a wide range of mathematical problems in phylogenetics (Rhodes and Sullivant 2012; Allman et al. 2013; Allman and Rhodes 2003, 2005, 2006, 2007, 2008, 2009; Allman et al. 2014).

A *split* is a partition of the set of taxa into two non-empty parts. We can construct a flattening for any split. The rows of the flattening are indexed by all $r^{|A|}$ ways of assigning a state to the taxa in *A* and the columns are indexed by all $r^{|B|}$ ways of assigning a state to the taxa in *B*. Each entry equals a term $p_{i_1i_2\cdots i_n}$ with each state $i_k$ determined by the row index if $k \in A$ and by the column index if $k \in B$. We denote this matrix $\textrm{flat}_{A|B}$. The matrix in (2) is $\textrm{flat}_{\{1,2\}|\{3,4\}}$ and corresponds to the split $\{1,2\} | \{3,4\}$. The flattening for split $\{1,3\}|\{2,4\}$ is

$$\begin{aligned} \textrm{flat}_{\{1,3\}|\{2,4\}} = \left[ \begin{matrix}p_{\tiny {\texttt{R}}\tiny {\texttt{R}}\tiny {\texttt{R}}\tiny {\texttt{R}}} &{} p_{\tiny {\texttt{R}}\tiny {\texttt{R}}\tiny {\texttt{R}}\tiny {\texttt{Y}}} &{} p_{\tiny {\texttt{R}}\tiny {\texttt{Y}}\tiny {\texttt{R}}\tiny {\texttt{R}}} &{} p_{\tiny {\texttt{R}}\tiny {\texttt{Y}}\tiny {\texttt{R}}\tiny {\texttt{Y}}} \\ p_{\tiny {\texttt{R}}\tiny {\texttt{R}}\tiny {\texttt{Y}}\tiny {\texttt{R}}} &{} p_{\tiny {\texttt{R}}\tiny {\texttt{R}}\tiny {\texttt{Y}}\tiny {\texttt{Y}}} &{} p_{\tiny {\texttt{R}}\tiny {\texttt{Y}}\tiny {\texttt{Y}}\tiny {\texttt{R}}} &{} p_{\tiny {\texttt{R}}\tiny {\texttt{Y}}\tiny {\texttt{Y}}\tiny {\texttt{Y}}} \\ p_{\tiny {\texttt{Y}}\tiny {\texttt{R}}\tiny {\texttt{R}}\tiny {\texttt{R}}} &{} p_{\tiny {\texttt{Y}}\tiny {\texttt{R}}\tiny {\texttt{R}}\tiny {\texttt{Y}}} &{} p_{\tiny {\texttt{Y}}\tiny {\texttt{Y}}\tiny {\texttt{R}}\tiny {\texttt{R}}} &{} p_{\tiny {\texttt{Y}}\tiny {\texttt{Y}}\tiny {\texttt{R}}\tiny {\texttt{Y}}} \\ p_{\tiny {\texttt{Y}}\tiny {\texttt{R}}\tiny {\texttt{Y}}\tiny {\texttt{R}}} &{} p_{\tiny {\texttt{Y}}\tiny {\texttt{R}}\tiny {\texttt{Y}}\tiny {\texttt{Y}}} &{} p_{\tiny {\texttt{Y}}\tiny {\texttt{Y}}\tiny {\texttt{Y}}\tiny {\texttt{R}}} &{} p_{\tiny {\texttt{Y}}\tiny {\texttt{Y}}\tiny {\texttt{Y}}\tiny {\texttt{Y}}} \end{matrix}\right] \end{aligned}$$

and the flattening for $\{1\}|\{2,3,4\}$ is

$$\begin{aligned} \textrm{flat}_{\{1\}|\{2,3,4\}} = \left[ \begin{matrix}p_{\tiny {\texttt{R}}\tiny {\texttt{R}}\tiny {\texttt{R}}\tiny {\texttt{R}}} &{} p_{\tiny {\texttt{R}}\tiny {\texttt{R}}\tiny {\texttt{R}}\tiny {\texttt{Y}}} &{} p_{\tiny {\texttt{R}}\tiny {\texttt{R}}\tiny {\texttt{Y}}\tiny {\texttt{R}}} &{} p_{\tiny {\texttt{R}}\tiny {\texttt{R}}\tiny {\texttt{Y}}\tiny {\texttt{Y}}} &{} p_{\tiny {\texttt{R}}\tiny {\texttt{Y}}\tiny {\texttt{R}}\tiny {\texttt{R}}} &{} p_{\tiny {\texttt{R}}\tiny {\texttt{Y}}\tiny {\texttt{R}}\tiny {\texttt{Y}}} &{} p_{\tiny {\texttt{R}}\tiny {\texttt{Y}}\tiny {\texttt{Y}}\tiny {\texttt{R}}} &{} p_{\tiny {\texttt{R}}\tiny {\texttt{Y}}\tiny {\texttt{Y}}\tiny {\texttt{Y}}} \\ p_{\tiny {\texttt{Y}}\tiny {\texttt{R}}\tiny {\texttt{R}}\tiny {\texttt{R}}} &{} p_{\tiny {\texttt{Y}}\tiny {\texttt{R}}\tiny {\texttt{R}}\tiny {\texttt{Y}}} &{} p_{\tiny {\texttt{Y}}\tiny {\texttt{R}}\tiny {\texttt{Y}}\tiny {\texttt{R}}} &{} p_{\tiny {\texttt{Y}}\tiny {\texttt{R}}\tiny {\texttt{Y}}\tiny {\texttt{Y}}} &{} p_{\tiny {\texttt{Y}}\tiny {\texttt{Y}}\tiny {\texttt{R}}\tiny {\texttt{R}}} &{} p_{\tiny {\texttt{Y}}\tiny {\texttt{Y}}\tiny {\texttt{R}}\tiny {\texttt{Y}}} &{} p_{\tiny {\texttt{Y}}\tiny {\texttt{Y}}\tiny {\texttt{Y}}\tiny {\texttt{R}}} &{} p_{\tiny {\texttt{Y}}\tiny {\texttt{Y}}\tiny {\texttt{Y}}\tiny {\texttt{Y}}} \end{matrix}\right] . \end{aligned}$$

An important property of flattenings from phylogenies is their rank. Suppose that *e* is an edge in a phylogeny. Removing *e* partitions the tree, and hence the set of leaves, into two parts, inducing a split *A*|*B* of the set of taxa. We say that *A*|*B* is the *split of the tree corresponding to edge e*. Allman and Rhodes (2007) (Proposition 11) proved that, under minor assumptions, if *A*|*B* is a split in the tree then the rank of $\textrm{flat}_{A|B}$ is at most *r*, while if *A*|*B* is *not* a split of the tree then the rank of $\textrm{flat}_{A|B}$ is at least $r^2$.

Because of this property, flattenings and their ranks have played a prominent role in the mathematics of phylogenetics, particularly with respect to the development and construction of *phylogenetic invariants* (Allman and Rhodes 2008, 2005; Pachter and Sturmfels 2004). Roughly speaking, a phylogenetic invariant for a tree is a function on vectors of pattern probabilities such as (1) which is zero when the probability distribution comes from that tree and non-zero otherwise. Invariants provide an elegant and distinctive means to infer single phylogenies or classes of phylogenies, although the approach does not appear to have reached its potential.

Several other phylogenetic methods are based on flattenings. In an original and influential chapter, Eriksson (2005) outlined a polynomial-time method for inferring phylogenies with few assumptions about the evolutionary process. The SVDQuartets method (Chifman and Kubatko 2014) uses flattenings to infer trees for subsets of four taxa, subsequently assembling these four-taxa trees into one for the complete set of taxa. The method is statistically consistent even in the presence of incomplete lineage sorting. Quartet-based approaches based on flattening have also been developed by Fernández-Sánchez and Casanellas (2016), and Casanellas et al. (2021). An excellent review of many of these developments is found in Allman et al. (2017).

The calculation of the *split scores* described in Allman et al. (2017) is justified by rank bounds of flattenings. Given a split (and *r*-state sequence data) the split score is defined using the sum of all except the largest *r* singular values, divided by the sum of all singular values. If the data exactly reflects the pattern probabilities of a tree containing that split then the fact that the flattening has rank *r* gives a split score of 0. For other splits, the score will be higher, the intuition being that the worse a split fits the tree, the higher its split score will be. We provide a theoretical foundation for this intuition by showing that a split has low rank if and only if the split belongs to a nearby tree.

We note that our expression for the rank of a flattening $\textrm{flat}_{A|B}$ in a binary tree can be derived as a direct consequence of Proposition 3.1 in Casanellas and Fernández-Sánchez (2011). Casanellas and Fernández-Sánchez use group representation theory to prove a more general result, revealing structure within the flattenings due to symmetries in the transition matrices.

In this paper we extend and complete the theorem of Allman and Rhodes and derive an exact formula for the rank of arbitrary flattenings of a tree for both binary and non-binary trees. Our result corrects a formula appearing in Eriksson (2005), see (Casanellas and Fernández-Sánchez 2011), remark 3.3. We show that the rank of $\textrm{flat}_{A|B}$ is given by $r^{\nu _T(A|B)}$, where $\nu _T(A|B)$ is the size of the smallest vertex cut separating *A* and *B* (defined below). If *T* is binary, then $\nu _T(A|B)$ equals the parsimony length $\ell _T(A|B)$ of the binary character separating *A* and *B*, a classical measure of fit between a character and a tree (Felsenstein 2004). In constrast to Casanellas and Fernández-Sánchez (2011) we identify exactly the (modest) constraints on parameters required for the rank result to hold (conditions (C1)–(C3) below).

Our most important contribution is the simplicity of our proof, which uses little more machinery than undergraduate linear algebra. Nevertheless, it demonstrates a fundamental link between combinatorial and algebraic approaches to phylogenetics, domains which have been mainly progressing in parallel. As a direct application we prove an exact formula connecting flattening rank and standard distances between trees (Theorem 9).

In the next section we present the notation and basic results needed to establish this result. We note that we generalise from characters to maps on arbitrary subsets of vertices, and define our concepts in this more general context. The main result is proved in a series of lemmas and propositions in the final section.

## Background and definitions

In this section we give a fairly minimal set of definitions sufficient to describe the main result. Excellent introductions to this material can be found in Felsenstein (2004), Semple and Steel (2003), Steel (2016).

### Trees and characters

#### Definition 1

An *unrooted phylogeny* is an acyclic, connected graph $T = (V,E)$ with leaf set (taxon set) *L*(*T*). We say that *T* is binary (full resolved) if every non-leaf vertex has degree three. In a *rooted phylogeny* $T = (V,E_\rho )$ one vertex is selected as the root $\rho$ and edges are directed away from $\rho$. The rooted phylogeny *T* is binary if every non-leaf vertex has out-degree 2.

#### Definition 2

A *character* is a function *f* from *L*(*T*) to a set of states $[r] = \{1,2,\ldots ,r\}$. We will consider a more general situation where *f* has a domain $\textrm{dom}(f)$ equal to any non-empty subset of *V*, not just *L*(*T*).

One example of a character is the map from *L*(*T*) to the the set of nucleotides given by a column of the aligned sequences. In this case, $r = 4$ and the four states correspond to *A*, *C*, *G* or *T*. In some situations, it can be advantageous to recode the four states as two: one for purines (*A* or *G*, coded as $\texttt{R}$) and pyrimidines (*C* or *T*, coded as $\texttt{Y}$), giving an example where $r=2$. With protein-coding sequence, it is common to define characters using the amino acids determined by triples of nucleotides (codons), giving characters with $r=20$. In fact *r* can become arbitrarily large in covarion or profile models since the set of states for these models is the product of the set of nucleotides (or proteins) and a set of rate or profile classes (Yourdkhani et al. 2021; Tuffley and Steel 1998; Galtier 2001).

#### Definition 3

The *length* of a function $F:V \rightarrow [r]$ is defined as

$$\begin{aligned} \ell _T(F) = |\left\{ \{u,v\} \in E: F(u) \ne F(v) \right\} | \end{aligned}$$

and the (parsimony) length of a function *f* with domain $\textrm{dom}(f) \subseteq V$ is the length of a minimum extension

$$\begin{aligned} \ell _T(f) = \min \{ \ell _T(F) : F|_{\textrm{dom}(f)} = f \}. \end{aligned}$$

The length of a function or character can be expressed equivalently using vertex and edge cuts (Semple and Steel 2003). For $E' \subseteq E$ or $V' \subseteq V$ we let $T \setminus E'$ and $T \setminus V'$ denote the graphs resulting from deleting $E'$ or $V'$ respectively. The length $\ell _T(f)$ equals the minimum cardinality of an edge cut $E' \subseteq E$ such that $f(u) = f(v)$ whenever *u* and *v* are in the same component of $T\setminus E'$. In a similar way, we let $\nu _T(f)$ denote the minimum cardinality of a vertex cut $V' \subseteq V$ such that $f(u) = f(v)$ whenever *u* and *v* are in the same component of $T \setminus V'$. Note that $V'$ can intersect $\textrm{dom}(f)$, and that for all *f* we have $\nu _T(f) \le \ell _T(f)$.

If *A* and *B* are disjoint subsets of *V* then we let $\ell _T(A|B)$ and $\nu _T(A|B)$ denote $\ell _T(f)$ and $\nu _T(f)$, where *f* equals the indicator function

$$\begin{aligned} f(x) = {\left\{ \begin{array}{ll} 0 &{} \text{ if } x\in A \\ 1 &{} \text{ if } x \in B. \end{array}\right. } \end{aligned}$$

Steel (1993); Semple and Steel (2003) observed that the parsimony length of a character with two states can be expressed in terms of the size of disjoint path sets by Menger’s theorem.

#### Proposition 4

Let $T = (V,E)$ be an unrooted phylogeny and let *A* and *B* be disjoint subsets of *V*. Then $\ell _T(A|B)$ equals the maximum cardinality of a set of edge-disjoint paths connecting vertices in *A* with vertices in *B*, while $\nu _T(A|B)$ equals the maximum cardinality of a set of vertex-disjoint paths connecting vertices in *A* with vertices in *B*.

Suppose that *T* is binary and that $p_1$ and $p_2$ are any two paths beginning and ending at leaves of *T*. We then have that $p_1$ and $p_2$ are edge disjoint if and only if they are vertex disjoint, and so obtain the following corollary of Proposition 4.

#### Corollary 5

Let $T = (V,E)$ be a binary phylogeny and suppose *A* and *B* are disjoint subsets of *L*(*T*). Then $\ell _T(A|B) = \nu _T(A|B)$.

**Fig. 2:**
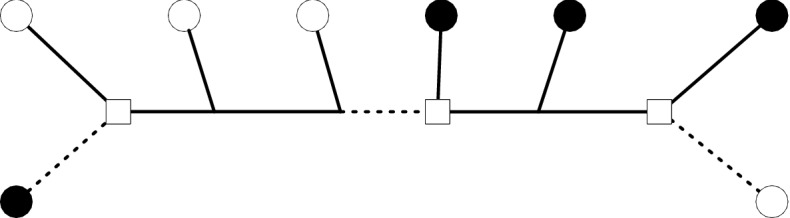
An example of a minimal edge cut (dotted edge) and a minimal vertex cut (square nodes) for a split *A*|*B* represented here using filled and unfilled circles

Figure 2 illustrates $\ell _T$ and $\nu _T$. Leaves in *A* and *B* are indicated using filled and unfilled leaf nodes. The dotted edges form a minimal edge cut while the square vertices form a minimal vertex cut.

**Fig. 3:**
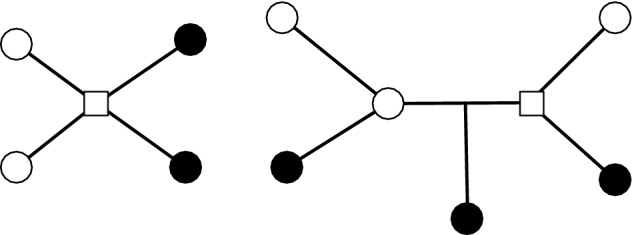
The tree on the left is an example where the minimum vertex cut (the central square node) is strictly smaller than a minimum edge cut. In the example on the right, the sets *A* and *B* are arbitrary disjoint subsets of vertices. Furthermore the minimum vertex cut (the square node and interior circle node) includes a node in $A \cup B$

Corollary 5 states that if *A* and *B* are sets of leaves in a binary tree *T* then $\ell _T(A|B) = \nu _T(A|B)$. Equality does not hold when *T* is not binary nor when *A* and *B* are not restricted to leaves (Fig. 3).

### Distances between trees

**Fig. 4:**
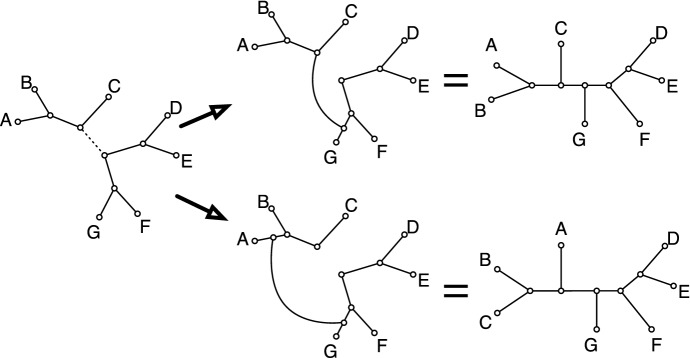
Two standard rearrangements for phylogenetic trees. In the first, subtree-prune-and-regraft (SPR) an edge in the tree (left) is removed and one of its endpoints is connected to a point in the opposite component (top middle) after which degree two vertices are surpressed. In the second, tree-bisection-and-regraft (TBR) an edge in the tree (left) is removed. A new edge which connects any two points in each component, and degree two vertices are surpressed. Every SPR operation is a TBR operation but not vice versa

Bryant (2004) showed that the parsimony score of a binary character (or split) can be expressed in terms of either distances based on rearrangements of phylogenetic trees (Fig. 4). In a subtree-prune-and-regraft (SPR) rearrangement an edge in the tree is removed and one endpoint of the edge is connected to an arbitrary location in the opposite component. In a tree-bisection-and-regraft (TBR) an edge is removed and a new edge is inserted between an arbitrary location in one component and an arbitrary location in the other component. By definition, any SPR rearrangement is also a TBR rearrangement, but there are TBR rearrangements which are not SPR rearrangements.

Both SPR and TBR rearrangements are extensively used in optimization and sampling algorithms for phylogenetic inference, and have been for some time (Swofford et al. 1996). The operations induce a natural edit distance on phylogenetic trees. Let $T_1$ and $T_2$ be two fully-resolved trees. The SPR-distance $d_{SPR}(T_1,T_2)$ is the smallest number of SPR rearrangements required to transform $T_1$ into $T_2$. The TBR-distance $d_{TBR}(T_1,T_2)$ is the smallest number of TBR rearrangements required to transform $T_1$ into $T_2$ (Allen and Steel 2001).

### The general Markov model of sequence evolution

We assume the *general Markov model* of sequence evolution, which we introduce here. The model was first proposed by Barry and Hartigan (1987). It is general enough that essentially all standard models for sequence evolution on a phylogeny with iid sites can be considered as special cases (Jayaswal et al. 2005).

Let $T = (V,E_\rho )$ be a rooted tree with root $\rho$. Let $\pi _\rho$ denote the root distribution and for each directed edge (*u*, *v*) we associate an $r \times r$ transition probability matrix $P_{uv}$. Let $X_v$ denote the random state associated with vertex *v* and, for $A \subseteq V$, let $X_A$ denote the joint random variable $X_a:a \in A$.

The joint probability that $X_v = F(v)$ for all $v \in V$ is defined

$$\begin{aligned} \pi (X_V = F) = \pi _\rho (F(\rho )) \prod _{(u,v) \in E_\rho } P_{uv} (F(u),F(v)). \end{aligned}$$

The marginal probabilities for maps $f:A \rightarrow [r]$ on some subset $A \subseteq V$ are then given by

$$\begin{aligned} \pi (X_A = f) = \sum _{F:F|_A = f} \pi (F). \end{aligned}$$

With this notation, the probability for a character $f:L(T) \rightarrow [r]$ is $\pi (X_{L(T)} = f)$.

In addition to the general Markov model we will be making the following, modest, assumptions about transition matrices:

1. The transition matrices $P_{uv}$ are non-singular.
2. $$\pi _\rho (i) >0$$ for all $$i \in [r]$$.
3. $P_{uv}(i,j)>0$ for all internal edges (*u*, *v*) and $i,j \in [r]$.

In the final section we show that these conditions are required for the main rank result to hold.

We will make use of the following consequences of (C1)–(C3), all of which can be found in the literature (e.g. Allman and Rhodes 2003; Steel et al. 1994; Steel 2016) or are proved directly from positivity and invertibility requirements.

#### Proposition 6

Suppose that conditions (C1), (C2) and (C3) are satisfied. Then

1. $\pi (X_v = i) >0$ for all $v \in V$ and $i \in [r]$.
2. For all $u,v \in V$ (not necessarily adjacent) the $r \times r$ matrix with entries $P_{uv} = \pi (X_v = j | X_u = i)$ is non-singular.
3. For all $u,v \in V$ the $r \times r$ matrix with entries $M_{ij} = \pi (X_u = i , X_v = j)$ is non-singular.
4. We can move the root to any internal vertex and update root distribution and transition matrices so that conditions (C1)–(C3) are still satisfied and all character probabilities are unchanged.

Conditional independence for the variables $X_v$ is, as with any graphical model, determined by cuts in the graph. More specifically, if $A_1,A_2,\ldots ,A_k$ are the components of $T\setminus \{v\}$ then $X_{A_1},X_{A_2},\ldots ,X_{A_k}$ are conditionally independent, given $X_v$, see (Steel 2016, Lemma 7.1). The following Proposition is a direct consequence of this result

#### Proposition 7

If $V' \subseteq V$ and $A_1,A_2,\ldots ,A_k$ are the components of $T\setminus \{V'\}$ then $X_{A_1},X_{A_2},\ldots ,X_{A_k}$ are conditionally independent, given $X_{V'}$.

#### Proof

Apply Lemma 7.1 of Steel (2016) with respect to some $v \in V'$ and then apply the result recursively on the components. $\square$

### The main theorems

Let $T=(V,E)$ be a phylogeny and suppose that *A* and *B* are disjoint subsets of *V*. We let $\textrm{flat}_{A|B}$ denote the matrix with rows indexed by maps $f_A:A \rightarrow [r]$, columns indexed by maps $f_B:B \rightarrow [r]$ and $(f_A,f_B)$ entry given by

$$\begin{aligned} \textrm{flat}_{A|B}(f_A,f_B) = \pi (X_A = f_A, X_B = f_B).\end{aligned}$$

Assumptions (C1)–(C3) imply that $\textrm{flat}_{A|B}$ is an $r^{|A|} \times r^{|B|}$ matrix with entries which are strictly positive and sum to 1.

#### Theorem 8

Let $T = (V,E)$ be a phylogeny with root distribution and transition matrices satisfying (C1)–(C3). If *A* and *B* are disjoint subsets of *V* then

$$\begin{aligned} \textrm{rank}(\textrm{flat}_{A|B}) = r^{\nu _T(A|B)}. \end{aligned}$$

If *T* is binary and *A* and *B* subsets of *L*(*T*) then

$$\begin{aligned} \textrm{rank}(\textrm{flat}_{A|B}) = r^{\ell _T(A|B)}. \end{aligned}$$

As a corollary of Theorem 8 we obtain the result of Allman and Rhodes (2007) that if *A*|*B* is a split in the tree then the rank of $\textrm{flat}_{A|B}$ is *r*, while if *A*|*B* is not a split in the tree then the rank of $\textrm{flat}_{A|B}$ is at least $r^2$.

Combining Theorem 8 with results of Bryant (2004) we connect the rank of flattenings with neighborhoods of trees.

#### Theorem 9

Let $T = (V,E)$ be a binary phylogeny with root distribution and transition matrices satisfying (C1)–(C3). Let *A*|*B* be a split and let $T_1$ and $T_2$ be the closest trees to *T* which contain the split *A*|*B*, with respect to SPR and TBR distances respectively. Then

$$\begin{aligned} rank(flat_{A|B}) = r^{d_{SPR}(T,T1) + 1} = r^{d_{TBR}(T,T_2) + 1} \end{aligned}$$

## Proof of theorem 8

To prove the main theorem we first show that $r^{\nu _T(A|B)}$ provides an *upper bound* for the rank of $\textrm{flat}_{A|B}$ and then show that this upper bound is actually achieved.

### Lemma 10

Let $T = (V,E)$ be a phylogeny and suppose that *A* and *B* are disjoint subsets of *V*. Then

$$\begin{aligned} \textrm{rank}(\textrm{flat}_{A|B}) \le r^{\nu _T(A|B)}. \end{aligned}$$

### Proof

Let *C* be a minimum cardinality vertex cut of *T* such that each component of $T \setminus C$ contains vertices from at most one of *A* or *B*. By Proposition 7$X_{A \setminus C}$ and $X_{B \setminus C}$ are conditionally independent given $X_C$. Hence for all maps $f_A:A \rightarrow [r]$ and $f_B:B \rightarrow [r]$ we can factor $\pi (X_A = f_A, X_B = f_B)$ as

$$\begin{aligned} \pi (X_A = f_A, X_B = f_B)&= \sum _{f_C} \pi (X_A = f_A, X_B = f_B|X_C = f_C ) \pi (X_C = f_C) \\&= \sum _{f_C} \pi (X_A = f_A |X_C = f_C) \pi (X_B = f_B, X_C = f_C) \\ \end{aligned}$$

Define the $r^{|A|} \times r^{|C|}$ matrix *R* by

$$\begin{aligned} R(f_A,f_C) = \pi (X_A = f_A |X_C = f_C), \end{aligned}$$

and the $r^{|C|} \times r^{|B|}$ matrix *S* by

$$\begin{aligned} S(f_C,f_B) = \pi (X_B = f_B, X_C = f_C). \end{aligned}$$

Then

$$\begin{aligned} \textrm{flat}_{A|B} = RS \end{aligned}$$

and

$$\begin{aligned} \textrm{rank}(\textrm{flat}_{A|B}) \le \textrm{rank}(R) \le r^{|C|} = r^{\nu _T(A|B)}.\end{aligned}$$

$$\square $$

To illustrate, consider the tree in Fig. 2. The sets *A* and *B* are indicated by filled and unfilled leaves respectively. Removing the three marked vertices separates all leaves in *A* from leaves in *B*. Hence $\nu _T(A|B) \le 3$ and $\textrm{rank}(\textrm{flat}_{A|B}) \le r^{\nu _T(A|B)}$. We note that the formula in Eriksson (2005) gives an incorrect generic rank of $r^4$ for this flattening, as observed by Casanellas and Fernández-Sánchez (2011).

We now work towards finding a matching lower bound for Lemma 10. We start by demonstrating that the rank of $\textrm{flat}_{A|B}$ does not increase if we marginalise over a subset of the variables.

### Lemma 11

Let *A* and *B* be disjoint subsets of *V* and suppose $A' \subseteq A$ and $B' \subseteq B$. Then

$$\begin{aligned} \textrm{rank}(\textrm{flat}_{A'|B'}) \le \textrm{rank}(\textrm{flat}_{A|B}). \end{aligned}$$

### Proof

The idea behind this proof is to show that $\textrm{flat}_{A'|B'}$ can be expressed as the product of $\textrm{flat}_{A|B}$ with two other matrices, from which the rank inequality follows.

For any $f_{A'}:A' \rightarrow [r]$ and $f_{B'}:B' \rightarrow [r]$ we have

$$\begin{aligned} \textrm{flat}_{A'|B'}(f_{A'},f_{B'})&= \pi (X_{A'} = f_{A'}, X_{B'} = f_{B'}) \\&= \sum _{\begin{array}{c} g_A:A \rightarrow [r] \\ g_A|_{A'} = f_{A'} \end{array}} \sum _{\begin{array}{c} g_B:B \rightarrow [r] \\ g_B|_{B'} = f_{B'} \end{array}} \pi (X_A = g_A, X_B = g_B) \\&= \sum _{\begin{array}{c} g_A:A \rightarrow [r] \\ g_A|_{A'} = f_{A'} \end{array}} \sum _{\begin{array}{c} g_B:B \rightarrow [r] \\ g_B|_{B'} = f_{B'} \end{array}} \textrm{flat}_{A|B}(g_A,g_B). \end{aligned}$$

Hence there is an $r^{|A'|} \times r^{|A|}$ $0\!-\!1$ (binary) matrix $U_A$ and a $r^{|B'|} \times r^{|B|}$ $0\!-\!1$ matrix $U_B$ such that

$$\begin{aligned}\textrm{flat}_{A'|B'} = U_A \textrm{flat}_{A|B} U_B^T\end{aligned}$$

and $$\textrm{rank}(\textrm{flat}_{A'|B'}) \le \textrm{rank}(\textrm{flat}_{A|B})$$. $$\square $$

The next Lemma establishes Theorem 8 in the extremal case that $\nu _T(A|B) = |A| = |B|$. This is where the bulk of the work proving the main theorem is carried out.

### Lemma 12

Suppose that $T=(V,E)$, *A* and *B* satisfy the conditions of Theorem 8. If $|A| = |B| = m$ and there are *m* disjoint paths connecting elements of *A* to elements of *B* then $\textrm{rank}(\textrm{flat}_{A|B}) = r^m$.

### Proof

We prove the result by induction on *m*.

For the base case suppose that $m=1$, $A = \{a\}$ and $B = \{b\}$. Then

$$\begin{aligned}\textrm{flat}_{A|B}(i,j) = \pi (X_a = i,X_B = j)\end{aligned}$$

so $\textrm{flat}_{A|B}$ is full rank $r = r^m$ by Proposition 6.

**Fig. 5:**
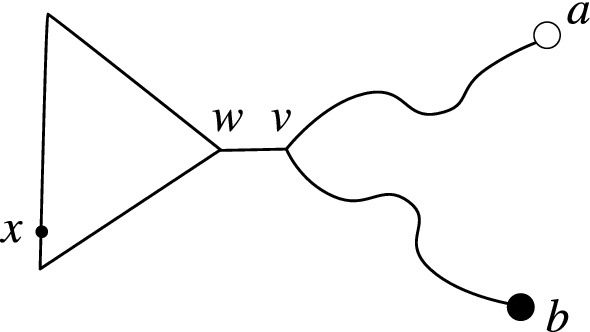
Labelling for the induction step for Lemma 12

Next, assume that the result holds whenever $|A| = |B| = m$. Suppose that there is a collection ${\mathcal {P}}'$ of $m+1$ vertex disjoint paths connecting elements of *A* and *B*, where $|A| = |B| = m+1$. There is a path *p* which is separated from the rest of the paths in ${\mathcal {P}}'$ by removal of a single edge in the tree. This can be seen by fixing an arbitrary vertex *u* and then taking *p* to be the path which is furthest from *u*. We let *v* be the vertex on *p* which is closest to *u*, and let $e = \{v,w\}$ denote the first edge on the path from *v* to *u* (Fig. 5).

Let $a \in A$ and $b \in B$ be the endpoints of the path *p* and let $A' = A \setminus \{a\}$, $B' = B \setminus \{b\}$. Given any function $f:A \rightarrow [r]$ or $g:B \rightarrow [r]$ we use $f'$ and $g'$ to denote the restriction of *f* to *A* or *g* to $B'$.

Define the matrices *F*, *G* by

$$\begin{aligned} F(i,k)&= \pi (X_a = i|X_v = k) \\ G(k,j)&= \pi (X_v = k,X_b = j) \end{aligned}$$

and for each $k \in [r]$ define the $r^m\times r^m$ matrix $H^{(k)}$ by

$$\begin{aligned} H^{(k)}(f',g')&= \pi (X_{A'} = f',X_{B'}=g'|X_v = k). \end{aligned}$$

We consider the tree to be rooted at *v*. The matrices $F,G,H^{(k)}$ provide a decomposition of $\textrm{flat}_{A|B}$:

$$\begin{aligned} \textrm{flat}_{A|B} (f,g)&= \sum _{k=1}^r F(f(a),k) H^{(k)}(f',g') G(k,g(b)) \end{aligned}$$

which we rewrite using the Kronecker product ($\otimes$) as

3 $$\begin{aligned} \textrm{flat}_{A|B}&= (F \otimes I_{r^{m-1}}) \left[ \begin{matrix}H^{(1)} &{} &{} &{} \\ {} &{} H^{(2)} &{} &{} \\ {} &{} &{} \ddots &{} \\ {} &{} &{} &{} H^{(r)} \end{matrix}\right] (G \otimes I_{r^{m-1}}). \end{aligned}$$

The matrices *F* and *G* are both non-singular, by Proposition 6. Furthermore, for each *k* the matrix $H^{(k)}$ equals the flattening matrix $\textrm{flat}_{A|B}$ but with respect to root *w* and root distribution

$$\begin{aligned} \pi _w(i) = P_{vw}(k,i) \end{aligned}$$

which is strictly positive for all *i* by (C3). By the induction hypothesis, each matrix $H^{(k)}$ is also non-singular. It follows from standard properties of the Kronecker product (Horn and Johnson 1991, Chapter 4) that all three matrices in the product (3) are non-singular, so that $\textrm{flat}_{A|B}$ is also non-singular. $\square$

We can now prove the main theorem.

### Proof

(Theorem 8) Suppose that *A* and *B* are disjoint subsets of *V*. From Lemma 10 we have

$$\begin{aligned} \textrm{rank}(\textrm{flat}_{A|B}) \le r^{\nu _T(A|B)}. \end{aligned}$$

By Proposition 4 there are $\nu _T(A|B)$ vertex disjoint paths connecting vertices in *A* to vertices in *B*. Let ${\hat{A}}$ and ${\hat{B}}$ be the endpoints of these paths, so $|{\hat{A}}| = |{\hat{B}}| = \nu _T({\hat{A}}|{\hat{B}}) = \nu _T(A|B)$. By Lemma 12 we have $\textrm{rank}(\textrm{flat}_{{\hat{A}}|{\hat{B}}}) = r^{\nu _T(A|B)}$ and by Lemma 11 we have

$$\begin{aligned} \textrm{rank}(\textrm{flat}_{A|B}) \ge \textrm{rank}(\textrm{flat}_{{\hat{A}}|{\hat{B}}}) = r^{\nu _T(A|B)}. \end{aligned}$$

The second part of the theorem follows from Corollary 5. $\square$

Theorem 9 follows as a corollary of Theorem 8 and Theorem 5.2 of Bryant (2004).

We note that none of the conditions (C1)–(C3) on the root distribution and transition matrices can be eliminated. For example, consider the four taxa tree in Figure 1, a case studied in detail by Allman and Rhodes (2006). The joint probability distribution for $X_1,X_2,X_3,X_4$ is

$$\begin{aligned}{} & {} \pi (X_1=a,X_2=b,X_3=c,X_4=d) \\{} & {} \quad = \sum _i \sum _j P_{u1}(i,a) P_{u2}(i,b) \pi _{\rho }(i) P_{uv}(i,j) P_{v3}(j,c) P_{v4}(j,d) .\end{aligned}$$

Gather terms, we obtain a decomposition

$$\begin{aligned} \textrm{flat}_{\{1,3\}|\{2,4\}} = UDV \end{aligned}$$

where *U* and *V* are $r^2 \times r^2$ matrices and *D* is a diagonal matrix with diagonal entries

$$\begin{aligned} D_{ij;ij} = \pi _{\rho }(i) P_{uv}(i,j). \end{aligned}$$

From here we see that if there is *i* such that $\pi _\rho (i) = 0$, or *ij* such that $P_{uv}(i,j) = 0$, then

$$\begin{aligned} \textrm{rank}(\textrm{flat}_{\{1,3\}|\{2,4\}} ) < r^2. \end{aligned}$$

The example can be extended directly to any tree with more than four taxa.
